# Case Report: An enlarging left ventricular mass during anticoagulation in a patient receiving almonertinib after thoracic radiotherapy: diagnostic uncertainty and individualized management

**DOI:** 10.3389/fcvm.2026.1953416

**Published:** 2026-09-30

**Authors:** Bin He, Yu Tan, Fangyuan Kong, Hua Li, Ling Zhang

**Affiliations:** 1 Department of Oncology and Radiotherapy, Chengdu Qingbaijiang District People’s Hospital, Chengdu, Sichuan, China; 2 School of Clinical Medicine, Chengdu Medical College, Chengdu, China; 3 Department of Oncology, General Hospital of Western Theater Command, People’s Liberation Army, Chengdu, Sichuan, China

**Keywords:** almonertinib, cardio-oncology, direct oral anticoagulant, left ventricular thrombus, non-small cell lung cancer

## Abstract

Distinguishing a left ventricular thrombus from a cardiac tumor is challenging when a large mass arises at an atypical non-apical site, particularly in a patient with cancer. We describe a 37-year-old man with epidermal growth factor receptor-mutant non-small cell lung cancer receiving almonertinib who developed a broad-based posterior wall left ventricular mass while cancer imaging remained stable. At detection, the reported left ventricular ejection fraction estimate was 55%, and no regional wall motion abnormality was documented. Cardiac CT showed no calcification or appreciable arterial- or delayed-phase enhancement, and Doppler echocardiography showed no internal flow. Low-mechanical-index contrast-enhanced echocardiography showed no appreciable internal perfusion in the contemporaneous examination report; later archived images also showed persistent absence of perfusion, although early-phase cine loops were not retained for retrospective reassessment. These concordant avascular features supported the presence of a thrombus but were not diagnostic. Cardiac magnetic resonance was recommended but declined because of financial concerns, and histopathology was unavailable. After approximately 9 months of prescribed edoxaban 60 mg once daily with reported adherence, the maximum linear dimension increased from 39.7 to 51.0 mm. A posterior wall regional wall motion abnormality was first documented 5 months after mass detection and therefore could not explain the lesion already present at detection. Edoxaban levels and edoxaban-calibrated anti-factor Xa activity were not measured, and human interaction data for almonertinib and edoxaban were unavailable. This case highlights the need to keep the diagnosis qualified and to reassess alternative pathology and anticoagulant exposure when an imaging-supported left ventricular thrombus follows an unexpected course.

## 1 Introduction

Third-generation epidermal growth factor receptor tyrosine kinase inhibitors (EGFR-TKIs), including osimertinib and almonertinib (aumolertinib), have improved disease control in advanced EGFR-mutant non-small cell lung cancer (NSCLC) (1, 2). As treatment continues for longer periods, cardiovascular problems may arise even when the cancer is controlled. Reduced left ventricular ejection fraction (LVEF) and heart failure have been reported with osimertinib (3). Almonertinib trials reported a generally manageable safety profile but provided limited detail about specific cardiac events (2, 4).

A left ventricular thrombus (LVT) often forms in akinetic or severely hypokinetic myocardium, where reduced ventricular function and blood stasis increase thrombotic and embolic risk (5). In cancer, an intracardiac mass must also be distinguished from cardiac metastasis or another tumor, making multimodal imaging important (6). Anticoagulation is the main treatment for established LVT, but management is less certain when a presumed thrombus persists or enlarges (5, 7). We describe an atypically located posterior wall mass that imaging favored as a thrombus but did not definitively establish, with enlargement during anticoagulation and later regional ventricular dysfunction.

## 2 Case description

### 2.1 Oncologic history and treatment

In March 2020, a 32-year-old Chinese man who worked as a fruit farmer presented with left-sided low back pain. He had chronic hepatitis B and had taken entecavir for 10 years. He had no known cardiovascular disease, hypertension, diabetes, dyslipidemia, obesity, chronic kidney disease, or previous thrombosis. He did not smoke or drink alcohol. Chest computed tomography (CT) showed a 2.7 cm × 2.3-cm mass in the dorsal segment of the left lower lobe. Separate nodules were present in another ipsilateral lobe. The left hilar and mediastinal lymph nodes were enlarged, and the left pleura was thickened without a malignant effusion. CT-guided biopsy confirmed lung adenocarcinoma. Next-generation sequencing of the tumor-tissue specimen identified an EGFR exon 19 variant (c.2239_2248TTAAGAGAAG > C; p.L747_A750delinsP; variant allele frequency, 0.21%). Cranial and abdominal imaging showed no distant metastases. The disease was staged as cT4N2M0, stage IIIB, according to the eighth edition of the American Joint Committee on Cancer system (8).

Figure 1 and Supplementary Table S1 summarize the clinical course. From March to May 2020, the patient received intensity-modulated radiotherapy (IMRT) to the primary lung lesion and involved mediastinal nodes (55 Gy in 25 fractions, 6-MV photons). Retrospective re-review of the original treatment plan confirmed that all reported dose metrics were physical doses from the single thoracic IMRT plan, without EQD2 conversion or summation with a second radiotherapy plan. The whole heart and cardiac substructures [left ventricle (LV), left anterior descending coronary artery (LAD), and left circumflex coronary artery (LCx)] were retrospectively delineated on the planning CT for dose-volume histogram extraction. Whole-heart Dmax (maximum dose received by a structure) and Dmean (mean dose to a structure) were 62.17 and 15.02 Gy, respectively; V5, V10, V20, V30, and V40 were 49.71%, 44.81%, 32.10%, 25.12%, and 10.49%, respectively. LV Dmax and Dmean were 47.14 and 3.27 Gy, respectively. LAD Dmax and Dmean were 55.15 and 30.30 Gy, respectively; V20 and V40 were 72.10% and 37.65%, respectively. LCx Dmax and Dmean were 59.85 and 47.98 Gy, respectively; V20, V30, and V40 were 100%, 100%, and 85.0%, respectively. The whole-heart Dmax above the 55-Gy prescription arose within this original thoracic plan because the primary target directly abutted the heart and the high-dose gradient traversed adjacent cardiac structures; it did not reflect overlap with the later rib-radiotherapy plan. The relatively low LV mean dose reflected limited irradiation of the majority of the LV despite greater exposure of the adjacent left atrial/coronary region. Chemotherapy with pemetrexed 500 mg/m^2^ and carboplatin at an area under the curve of 5 began on 7 April 2020, concurrently with thoracic radiotherapy. Treatment was administered every 3 weeks for five cycles, with subsequent cycles continuing after radiotherapy. No grade 3 or higher treatment-related adverse event was recorded. He then started erlotinib 150 mg once daily. In November 2020, an eighth-rib metastasis was treated with 30 Gy in 10 fractions.

**Figure 1:**
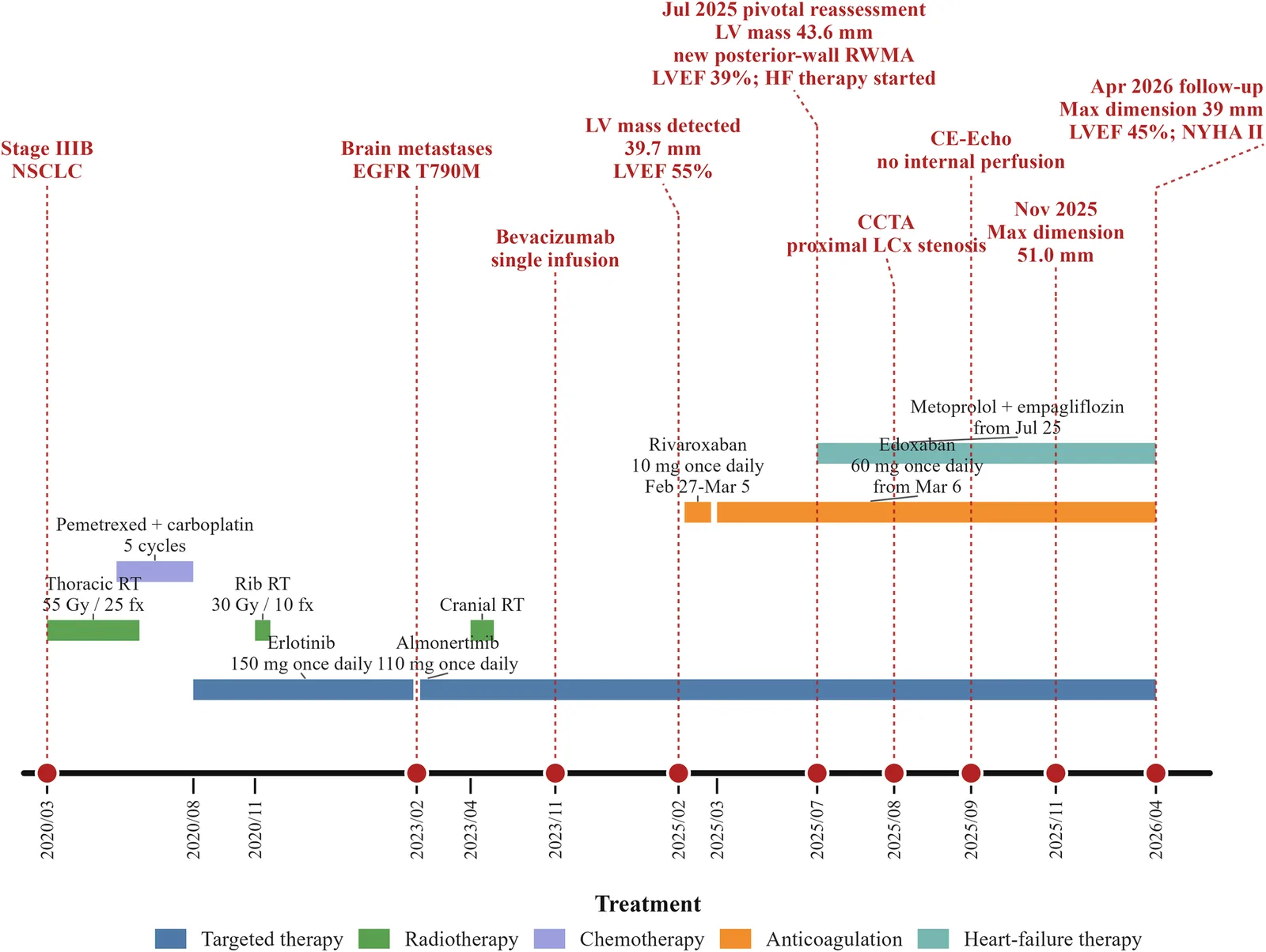
Schematic clinical timeline of oncological treatment and cardiovascular events. The timeline shows thoracic radiotherapy and five cycles of pemetrexed plus carboplatin, with chemotherapy initiated during radiotherapy and continued thereafter, followed by erlotinib beginning after completion of chemotherapy. Palliative eighth-rib radiotherapy (30 Gy in 10 fractions) was delivered in November 2020. After development of brain metastases and detection of EGFR T790M in early 2023, erlotinib was replaced with almonertinib. A left ventricular mass was detected in February 2025. The pivotal July 2025 reassessment showed interval enlargement of the mass to 43.6 mm, a newly documented posterior wall regional wall motion abnormality, a reported LVEF estimate of 39%, and initiation of metoprolol succinate and empagliflozin. Rivaroxaban 10 mg once daily was administered from 27 February to 5 March 2025, and edoxaban 60 mg once daily was started on 6 March. Subsequent evaluation included CCTA in August 2025 and CE-Echo in September 2025. The maximum linear mass dimension was 51.0 mm in November 2025 and 39 mm at follow-up in April 2026. Horizontal spacing is schematic and is not proportional to elapsed time. All LVEF values shown are clinically reported M-mode/Teichholz-derived estimates. CCTA, coronary computed tomography angiography; CE-Echo, contrast-enhanced echocardiography; fx, fractions; HF, heart failure; LCx, left circumflex coronary artery; LVEF, left ventricular ejection fraction; NSCLC, non-small cell lung cancer; NYHA, New York Heart Association; RT, radiotherapy; RWMA, regional wall motion abnormality.

The disease remained stable until early 2023. The patient then developed headache, numbness, and weakness. Brain magnetic resonance imaging showed multiple metastases. Plasma circulating tumor DNA next-generation sequencing detected an acquired EGFR T790M variant at a variant allele frequency of 0.16%. Erlotinib was replaced with almonertinib 110 mg once daily. Cranial radiotherapy was delivered in April 2023 with a planned gross tumor volume dose of 60 Gy. Later that year, intermittent right-sided headache and vomiting were attributed to cerebral edema. Symptoms improved with mannitol 0.25 g/kg every 6–8 h and dexamethasone 4 mg every 6 h. He also received one 300-mg infusion of bevacizumab on 16 November 2023. The thoracic and intracranial disease remained radiographically stable afterward (Figure 2 and Supplementary Figure S2).

**Figure 2:**
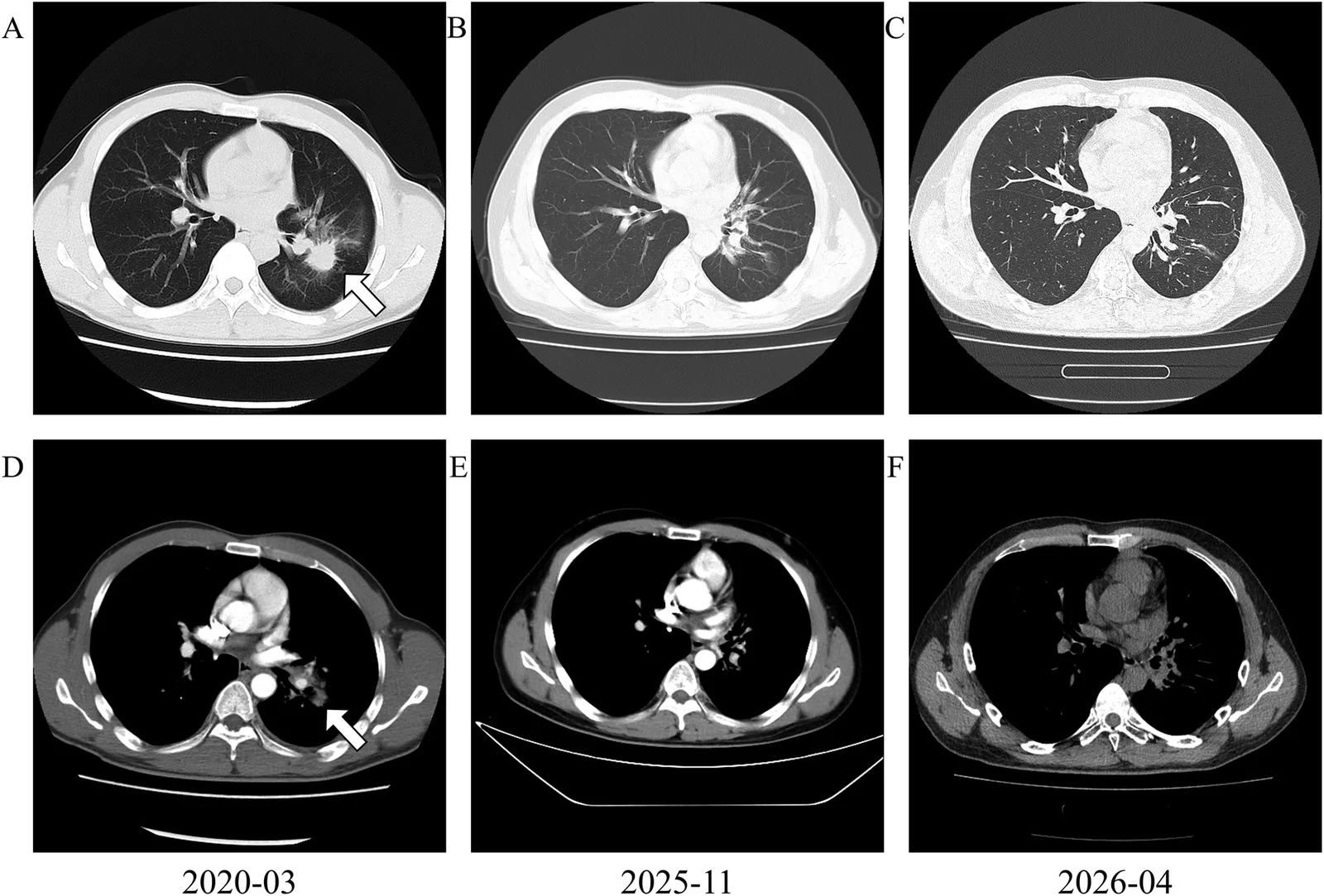
Serial thoracic computed tomography findings. **(A,D)** Images obtained in March 2020. The lung window shows the primary lesion in the dorsal segment of the left lower lobe (A, arrow). The contrast-enhanced mediastinal window shows left hilar and mediastinal nodal disease (D, arrow). **(B,E)** Corresponding images obtained in November 2025 show marked reduction of the primary and nodal lesions, with residual treatment changes. **(C,F)** Non-contrast images obtained in April 2026 show no clear interval progression of thoracic disease. CT, computed tomography.

### 2.2 Diagnostic assessment

On 18 February 2025, when the patient was 37 years old, contrast-enhanced chest CT showed a new posterior left ventricular filling defect without definite enhancement. No calcification was evident on the available non-contrast images. The patient reported dyspnea and fatigue only after climbing three to four flights of stairs and had no volume overload. His weight was 69 kg. Serum creatinine was 61 µmol/L. The estimated glomerular filtration rate was approximately 121 mL/min/1.73 m^2^ using the 2009 Chronic Kidney Disease Epidemiology Collaboration (CKD-EPI) creatinine equation. Baseline albumin, total bilirubin, and international normalized ratio (INR) were within reference ranges; platelets were 103 × 10^9^/L. Troponin, natriuretic peptide, and lipid levels were normal. Abdominal imaging showed a smooth liver contour, normal liver size and morphology, a normal-sized spleen, and no ascites or imaging evidence of portal hypertension. No clinical or imaging evidence of cirrhosis was identified, so no Child–Pugh class was assigned. Apart from almonertinib, no concomitant antiplatelet agent or other recorded prescription, over-the-counter, herbal, or dietary product likely to materially alter anticoagulant exposure was identified.

LVEF was calculated using the Teichholz formula from M-mode-derived linear left ventricular dimensions. On 26 February, a transthoracic echocardiogram (TTE) showed an irregular, relatively fixed, broad-based posterior wall mass measuring 39.7 mm × 13.4 mm × 30.9 mm without internal Doppler flow (Figure 3A). The reported LVEF estimate was 55%, and no regional wall motion abnormality was documented. Because the diagnosis was initially uncertain and bleeding risk was a concern in the setting of mild thrombocytopenia and chronic hepatitis B, rivaroxaban 10 mg once daily was started empirically on 27 February. It was stopped on 5 March because of severe nausea and vomiting. This prophylactic-intensity dose is not a guideline-supported treatment regimen for established LVT, and the 7-day exposure did not constitute an adequate therapeutic trial. Edoxaban 60 mg once daily was started on 6 March. The patient reported no missed doses or subsequent interruptions.

**Figure 3:**
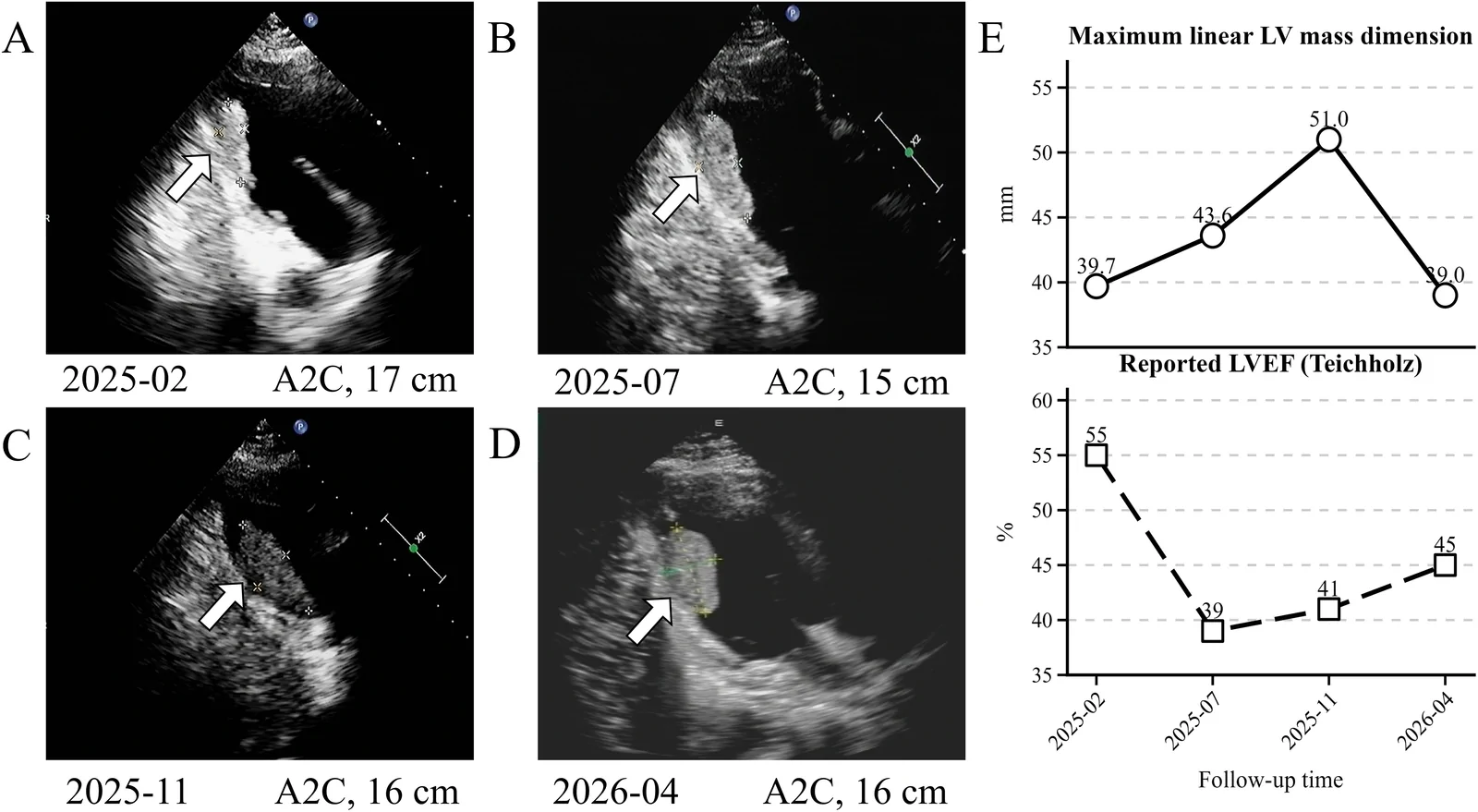
Serial transthoracic echocardiographic evolution of the left ventricular mass. Panels **A–D** show apical two-chamber (A2C) views; arrows indicate the left ventricular mass. The imaging depths were 17 cm in panel **A**, 15 cm in panel **B**, and 16 cm in panels **C** and **D**. **(A)** In February 2025, the broad-based posterior wall mass measured 39.7 mm × 13.4 mm × 30.9 mm; the reported M-mode/Teichholz-derived LVEF estimate was 55%, and no regional wall motion abnormality was documented. **(B)** In July 2025, the mass measured 43.6 mm × 18.3 mm × 19.9 mm. A posterior wall regional wall motion abnormality was newly documented, and the reported LVEF estimate was 39%. **(C)** In November 2025, the mass measured 51.0 mm × 20.0 mm × 27.0 mm during edoxaban therapy; the reported LVEF estimate was 41%. **(D)** In April 2026, the mass measured 39 mm × 19 mm × 29 mm. The maximum linear dimension was lower than in November 2025, whereas the other two measured dimensions were similar; therefore, no volumetric regression was inferred. The mass remained broad-based and relatively fixed, without internal Doppler flow, and the reported LVEF estimate was 45%, although ventricular motion remained reduced and dyssynchronous. **(E)** Serial plots summarize the maximum linear dimension of the left ventricular mass and the clinically reported M-mode/Teichholz-derived LVEF estimates. A2C, apical two-chamber view; LVEF, left ventricular ejection fraction.

A TTE on 25 July showed an irregular, relatively fixed mass measuring 43.6 mm × 18.3 mm × 19.9 mm without internal Doppler flow (Figure 3B). Electrocardiography showed sinus tachycardia at approximately 116 beats/min and non-specific changes; later tracings were similar. Hemoglobin and thyroid-function tests were normal, and no pulmonary embolism was identified on contemporaneous contrast-enhanced chest CT. A posterior wall regional wall motion abnormality and mild-to-moderate mitral regurgitation were first documented 5 months after detection of the mass. The M-mode/Teichholz-derived LVEF estimate was 39%. Metoprolol succinate 47.5 mg once daily and empagliflozin 10 mg once daily were introduced first as part of a staged, tolerability-oriented heart-failure strategy. A renin-angiotensin system inhibitor or angiotensin receptor-neprilysin inhibitor and a mineralocorticoid receptor antagonist were deferred because of the patient’s recent gastrointestinal intolerance and concern about early hemodynamic tolerability. The patient also declined additional medication at this time. Alanine aminotransferase later rose to 74 U/L without a documented change in bilirubin, albumin, INR, ascites, or encephalopathy. Edoxaban was continued without dose reduction or interruption, and alanine aminotransferase returned to the reference range after supportive treatment.

Oncologic imaging on 18 August showed stable lung and brain disease. Coronary CT angiography on 27 August showed moderate-to-severe proximal left circumflex coronary artery stenosis with a non-calcified plaque (Supplementary Figure S3). The maximum dimension of the left ventricular mass was approximately 4.1 cm, and its mean attenuation was 41.26 HU. The mass had no visible calcification, no clear continuity with the mitral annulus, and no obvious myocardial invasion. No appreciable enhancement was seen on arterial- or delayed-phase images. In September 2025, contrast-enhanced echocardiography was performed after peripheral intravenous administration of 1.5 mL of sulfur hexafluoride microbubbles (SonoVue). Low-mechanical-index contrast-specific imaging was used at a mechanical index of approximately 0.09. The contemporaneous examination report documented no appreciable internal perfusion of the mass after contrast administration. Early-phase contrast-specific cine loops were not archived and therefore could not be independently reassessed retrospectively. Later archived images at approximately 1 min 11 s and 2 min 23 s showed a persistent absence of perfusion (Figure 4). A quantitative mass-to-myocardium peak-intensity ratio was not available. These findings supported an avascular lesion and favored thrombus, but they were not diagnostic by themselves. Cardiac magnetic resonance was recommended, but the patient declined the examination because of financial concerns.

**Figure 4:**
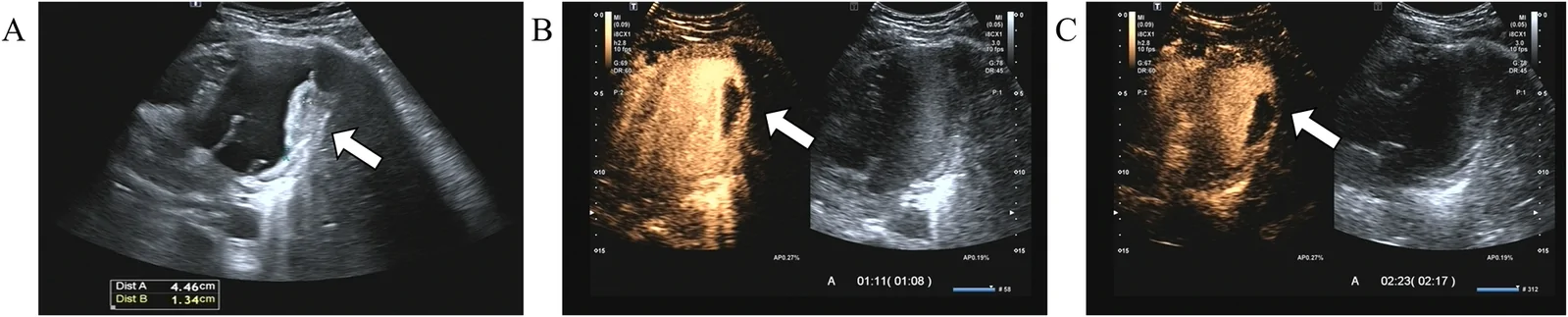
Contrast-enhanced echocardiographic characterization of the left ventricular mass in September 2025. **(A)** Conventional two-dimensional transthoracic echocardiography shows an irregular posterior wall mass measuring 4.46 × 1.34 cm. **(B,C)** After peripheral intravenous administration of 1.5 mL of sulfur hexafluoride microbubbles (SonoVue), low-mechanical-index contrast-specific imaging was performed at a mechanical index of approximately 0.09. The contemporaneous examination report documented no appreciable internal perfusion of the mass after contrast administration. Early-phase cine loops were not archived for retrospective reassessment. Later archived images at approximately 1 min 11 s **(B)** and 2 min 23 s **(C)** show the persistent absence of perfusion. Arrows indicate the mass. These findings supported an avascular lesion and favored a thrombus over a vascularized cardiac tumor, but they were not diagnostic by themselves. LV, left ventricle; TTE, transthoracic echocardiography.

### 2.3 Therapeutic intervention and follow-up

A TTE on 26 November showed a mass measuring 51.0 mm × 20.0 mm × 27.0 mm (Figure 3C) and an M-mode/Teichholz-derived LVEF estimate of 41%. The increase in the maximum linear dimension after approximately 9 months of prescribed treatment-dose edoxaban was considered atypical for a thrombus and prompted renewed assessment of the diagnosis and anticoagulant exposure. Edoxaban was continued without major or clinically relevant non-major bleeding. Edoxaban-calibrated anti-factor Xa activity and plasma edoxaban concentrations were not measured. Serial brain MRI showed no new acute or subacute ischemic lesion suggestive of systemic embolism. Oncology and cardiology teams jointly discussed cardiac magnetic resonance, a vitamin K antagonist, therapeutic low-molecular-weight heparin, continued edoxaban, and surgical thrombectomy. The patient declined surgery and long-term injections; cost and treatment burden were also considered. Edoxaban was therefore continued as an individualized conservative option. Cancer imaging remained stable, and carcinoembryonic antigen and D-dimer fluctuated without a consistent trend (Supplementary Figure S1).

In April 2026, chest CT again showed stable thoracic disease. A TTE showed a posterior wall mass measuring 39 mm × 19 mm × 29 mm (Figure 3D). The recorded maximum linear dimension was smaller than in November 2025, but the other two dimensions were similar. These linear measurements did not support a volumetric conclusion. The mass appeared more regular and broad-based but remained relatively fixed, with no internal Doppler flow. The M-mode/Teichholz-derived LVEF estimate was 45%, and fractional shortening was 22%; left ventricular wall motion remained reduced and dyssynchronous. The patient remained clinically stable with New York Heart Association class II symptoms and continued edoxaban and almonertinib.

## 3 Discussion

This case illustrates the diagnostic uncertainty created by a large posterior wall left ventricular mass with avascular imaging features but an atypical chronology. The lesion was first detected when the reported LVEF estimate was 55% and no regional wall motion abnormality had been documented. It then enlarged during prescribed treatment-dose anticoagulation. These two features required the working diagnosis of thrombus to be repeatedly reconsidered rather than treated as established.

The multimodal imaging findings favored an avascular thrombus but did not establish the diagnosis. Cardiac CT showed a 41.26-HU intracavitary mass without calcification, mitral-annular continuity, myocardial invasion, or arterial- or delayed-phase enhancement. Serial TTE showed no internal Doppler flow. Contrast-enhanced echocardiography adds real-time perfusion assessment when morphology is indeterminate; in a multicenter study, quantitative mass-to-myocardium peak-intensity analysis distinguished tumors from thrombi with an area under the curve of 0.977, 97.9% sensitivity, and 90.7% specificity (9). In this case, the contemporaneous report documented no internal perfusion and later archived images remained non-perfused, but early-phase cine loops and quantitative peak-intensity analysis were unavailable. Thus, the absence of perfusion supported an avascular thrombus but was not specific because poorly vascularized or necrotic tumors and technical factors can yield false-negative or indeterminate findings. Cardiac magnetic resonance provides more informative tissue characterization when uncertainty persists (6). Stable cancer, the absence of enhancement or invasion, and lack of calcification or mitral-annular continuity made metastasis, a vascularized primary tumor, and caseous mitral-annular calcification less likely, without excluding them. Organized hematoma and radiation-related fibrosis remained possible but lacked typical clinical context. Cardiac magnetic resonance was recommended but declined because of financial concerns, and histopathology was unavailable; the diagnosis therefore remained a presumptive, imaging-supported left ventricular thrombus.

The mass preceded regional left ventricular dysfunction. At detection in February 2025, the reported LVEF estimate was 55% and no regional wall motion abnormality was documented; posterior wall dysfunction was first documented 5 months later, so it could not have been the initial substrate. Proximal LCx stenosis may have contributed to later dysfunction, although its functional significance was not tested. Cancer-associated hypercoagulability remained compatible with a thrombus despite preserved systolic function (10), but the posterior wall location and enlargement to 51.0 mm over approximately 9 months of treatment-dose edoxaban were atypical (5, 6). These discordant features prompted renewed evaluation of neoplasm, another non-thrombotic mass, and the patient's anticoagulant exposure. The mass itself could also have contributed to the later regional mechanical abnormality, or the two findings may have represented parallel processes; causal direction remains uncertain. Rare malignancy-associated cardiovascular thrombosis has also been reported at unusual sites, including an ascending aortic thrombus causing intermittent coronary obstruction and acute coronary syndrome (11).

Previous cancer treatments were relevant but did not establish causation. Thoracic radiotherapy can lead to coronary and myocardial disease (12). In Radiation Therapy Oncology Group (RTOG) 0617, whole-heart V5 and V30 were associated with overall survival (13). In another NSCLC cohort, left anterior descending coronary artery dose was associated with major cardiac events and mortality (14). These population-level findings make the patient's prior exposure clinically relevant. Re-review of the original thoracic plan demonstrated substantial LCx exposure (Dmax 59.85 Gy, Dmean 47.98 Gy, V20 100%, V30 100%, and V40 85.0%) despite an LV mean dose of 3.27 Gy. This spatial pattern was consistent with the primary target lying close to the left atrial/LCx region while the majority of the LV received a limited dose. Even so, these findings do not show that radiotherapy caused the later LCx stenosis, ventricular dysfunction, or presumed LVT. The single bevacizumab infusion occurred approximately 15 months before detection of the left ventricular mass. Bevacizumab has been associated with arterial thromboembolic and ischemic events (15), but the long interval weakens a direct link in this case. Almonertinib was ongoing when the cardiac changes appeared. Published trials provide limited detail about specific cardiac events (2, 4). The coexisting LCx disease also makes attribution to almonertinib uncertain.

Anticoagulant intensity and exposure were considered separately. Rivaroxaban 10 mg once daily was used from 27 February to 5 March 2025 and was chosen with caution during diagnostic uncertainty because mild thrombocytopenia and chronic hepatitis B raised bleeding concerns. This prophylactic-intensity dose is not a guideline-supported treatment regimen for established LVT, and the 7-day exposure did not constitute an adequate therapeutic trial. Edoxaban 60 mg once daily began on 6 March with reported adherence. Edoxaban is a P-glycoprotein substrate, whereas almonertinib is a moderately sensitive CYP3A substrate in humans (16, 17). In rats, almonertinib increased rivaroxaban and apixaban exposure and altered transporter expression (18), but these data do not establish a human interaction with edoxaban and we could not find any human almonertinib-edoxaban pharmacokinetic studies. Because edoxaban levels and edoxaban-calibrated anti-factor Xa activity were not measured (19), systemic exposure could not be verified; enlargement therefore prompted reassessment of both diagnosis and drug exposure rather than attribution to edoxaban.

Management after interval enlargement was individualized. Cardiac magnetic resonance, a vitamin K antagonist, therapeutic low-molecular-weight heparin, continued edoxaban, and thrombectomy were considered (5). The patient declined cardiac magnetic resonance because of the cost. He also declined surgery, long-term injections, and additional heart failure medication at that stage. Treatment burden, cost, and quality of life were incorporated into shared decision-making. At later follow-up, one maximum linear mass dimension was smaller, while the other two dimensions were similar. This was not interpreted as volumetric regression or a drug-specific effect. Continued edoxaban was an individualized decision within a closely monitored multidisciplinary plan.

A strength of this case is the longitudinal multimodality imaging, which allowed the evolving mass phenotype, ventricular function, and diagnostic uncertainty to be reassessed over time. For an established LVT, repeat imaging at approximately 3 months is commonly used to reassess persistence and guide anticoagulation duration (5, 20, 21). Earlier reassessment at approximately 4–6 weeks may be reasonable when the location is atypical, diagnostic uncertainty persists, the mass enlarges, or ventricular function changes; lesions that persist during ongoing anticoagulation can be reassessed at approximately 6 months. TTE is practical for serial morphology and function, CE-Echo can add perfusion information, and cardiac magnetic resonance should be prioritized for unresolved tissue characterization (6, 9, 22, 23).

The limitations of this case include the absence of cardiac magnetic resonance and histopathology; the unavailability of early CE-Echo cine and quantitative perfusion analysis; and no edoxaban-calibrated anti-factor Xa activity, drug concentration, or pharmacogenetic data. Serial TTE examinations were performed and interpreted by the same senior echocardiographer; however, repeated or blinded measurements, intraobserver variability data, and standardized three-dimensional mass volumes were unavailable, precluding volumetric inference from linear dimensions. LVEF was derived using the M-mode Teichholz method, which is less reliable after a regional wall motion abnormality develops; global longitudinal strain was not prospectively acquired and archived routine images were unsuitable for reliable retrospective speckle tracking. These limitations affect tissue diagnosis, exposure assessment, and the precision of serial comparisons.

## 4 Conclusion

A posterior wall left ventricular mass in a patient with stable cancer showed concordant avascular features on CT, TTE, and contrast-enhanced echocardiography, which supported but did not prove the presence of a thrombus. The mass preceded the regional wall motion abnormality and enlarged during prescribed treatment-dose edoxaban. These findings required renewed evaluation of neoplasm, another non-thrombotic mass, and the patient's anticoagulant exposure. When cardiac magnetic resonance, histopathology, and drug-level testing are unavailable, the diagnosis should remain qualified and management should consist of scheduled multimodal reassessment and shared multidisciplinary decision-making.

## 5 Patient perspective

After his intracranial disease progressed in 2023, the patient decided to marry. He and his wife welcomed their first child approximately 1 year later. He said that his family gave him emotional support and helped him continue through prolonged and demanding cancer treatment. This case report was prepared in accordance with the CARE reporting guideline, and the completed checklist is provided in the Supplementary Material.

## Data Availability

The data supporting the conclusions of this article are available from the corresponding author on reasonable request, subject to patient privacy and applicable institutional requirements.
