# Supplementary material for: Case Report: An enlarging left ventricular mass during anticoagulation in a patient receiving almonertinib after thoracic radiotherapy: diagnostic uncertainty and individualized management
Source: Front Cardiovasc Med. 2026 Sep 30;13:1953416. doi: 10.3389/fcvm.2026.1953416 (PMC13661977; doi:10.3389/fcvm.2026.1953416)
Supplement: Supplementary file 1 [file Datasheet1.pdf]

CARE case report guidelines

CARE Checklist of information to include when writing a case report

(cc) BY-NC-ND

Topic	Item	Checklist item description	Reported on Line
Title	1	The diagnosis or intervention of primary focus followed by the words "case report"	Title page
Key Words	2	2 to 5 key words that identify diagnoses or interventions in this case report, including "case report"	14-15
Abstract (no references)	3a	Introduction: What is unique about this case and what does it add to the scientific literature?	17-18
	3b	Main symptoms and/or important clinical findings	18-32
	3c	The main diagnoses, therapeutic interventions, and outcomes	22-32
	3d	Conclusion—What is the main "take-away" lesson(s) from this case?	33-35
Introduction	4	One or two paragraphs summarizing why this case is unique (**may include references**)	37-49
Patient Information	5a	De-identified patient specific information.	52-62; 93-104
	5b	Primary concerns and symptoms of the patient.	52; 93-96
	5c	Medical, family, and psycho-social history including relevant genetic information	52-62; 260-262
	5d	Relevant past interventions with outcomes	63-91
Clinical Findings	6	Describe significant physical examination (PE) and important clinical findings.	93-120
Timeline	7	Historical and current information from this episode of care organized as a timeline	63; 376-393; Suppl. S1
Diagnostic Assessment	8a	Diagnostic testing (such as PE, laboratory testing, imaging, surveys).	93-144
	8b	Diagnostic challenges (such as access to testing, financial, or cultural)	138-144; 241-250
	8c	Diagnosis (including other diagnoses considered)	129-144; 167-199
	8d	Prognosis (such as staging in oncology) where applicable	61-62
Therapeutic Intervention	9a	Types of therapeutic intervention (such as pharmacologic, surgical, preventive, self-care)	63-91; 146-165
	9b	Administration of therapeutic intervention (such as dosage, strength, duration)	63-91; 108-165
	9c	Changes in therapeutic intervention (with rationale)	83-91; 108-165
Follow-up and Outcomes	10a	Clinician and patient-assessed outcomes (if available)	146-165; 260-264
	10b	Important follow-up diagnostic and other test results	146-165; 376-414
	10c	Intervention adherence and tolerability (How was this assessed?)	108-114; 146-165
	10d	Adverse and unanticipated events	108-114; 121-128; 149-156
Discussion	11a	A scientific discussion of the strengths AND limitations associated with this case report	233-250
	11b	Discussion of the relevant medical literature **with references**.	172-224
	11c	The scientific rationale for any conclusions (including assessment of possible causes)	190-232
	11d	The primary "take-away" lessons of this case report (without references) in a one paragraph conclusion	252-258
Patient Perspective	12	The patient should share their perspective in one to two paragraphs on the treatment(s) they received	260-264
Informed Consent	13	Did the patient give informed consent? Please provide if requested	266-269 Yes ☑ No ☐
