# Supplementary material for: Case Report: An enlarging left ventricular mass during anticoagulation in a patient receiving almonertinib after thoracic radiotherapy: diagnostic uncertainty and individualized management
Source: Front Cardiovasc Med. 2026 Sep 30;13:1953416. doi: 10.3389/fcvm.2026.1953416 (PMC13661977; doi:10.3389/fcvm.2026.1953416)
Supplement: Supplementary file 2 [file Supplementaryfile1.docx]

**Supplementary Table S1. Longitudinal clinical, imaging, and therapeutic course**

*LVEF values are clinically reported M-mode/Teichholz-derived estimates. Serial mass dimensions are two-dimensional linear measurements; the examinations were performed and interpreted by the same senior echocardiographer, but repeated or blinded measurements, intraobserver variability data, and standardized three-dimensional mass volumes were unavailable. No volumetric inference was made. Radiotherapy dose metrics are physical doses from the original single thoracic IMRT plan and were not EQD2-converted or summed with the later rib-radiotherapy plan.*

| **Date** | **Symptoms / clinical status** | **LV mass / cardiac imaging** | **Reported LVEF** | **Anticoagulation** | **Oncologic therapy / status** | **Key laboratory / other findings** |
| --- | --- | --- | --- | --- | --- | --- |
| Mar–Nov 2020 | Initial treatment for stage IIIB NSCLC; no known cardiovascular disease or prior thrombosis | No LV mass reported in the available records during initial oncologic treatment | — | None | Thoracic IMRT 55 Gy/25 fx (Mar–May); pemetrexed/carboplatin began Apr 7 concurrently, 5 cycles; erlotinib after chemotherapy; rib RT 30 Gy/10 fx (Nov). | Physical-dose DVH, single thoracic plan (no EQD2/plan sum): whole heart Dmax/Dmean 62.17/15.02 Gy; LV 47.14/3.27 Gy; LAD 55.15/30.30 Gy (V20 72.10%, V40 37.65%); LCx 59.85/47.98 Gy (V20/V30/V40 100%/100%/85.0%). |
| Feb 18–26, 2025 | Mild exertional dyspnea/fatigue after 3–4 flights of stairs; no volume overload | CT: new posterior-LV filling defect without definite enhancement/calcification. TTE: 39.7 × 13.4 × 30.9 mm broad-based posterior-wall mass; no internal Doppler flow; no RWMA documented | 55% | Rivaroxaban 10 mg once daily, Feb 27–Mar 5 | Almonertinib 110 mg once daily; thoracic and intracranial disease stable | Platelets 103 × 10⁹/L; creatinine 61 µmol/L; eGFR ≈121 mL/min/1.73 m² (2009 CKD-EPI); albumin, total bilirubin, INR within reference ranges |
| Jul 25, 2025 | Sinus tachycardia (~116 beats/min); no anemia or thyroid dysfunction; no PE identified on contemporaneous enhanced chest CT | TTE: 43.6 × 18.3 × 19.9 mm; new posterior-wall RWMA; mild-to-moderate mitral regurgitation | 39% | Edoxaban 60 mg once daily (from Mar 6; reported adherence) | Almonertinib continued | Metoprolol succinate 47.5 mg once daily and empagliflozin 10 mg once daily initiated |
| Aug–Sep 2025 | Diagnostic reassessment of enlarging LV mass | CCTA: mass ~4.1 cm, mean attenuation 41.26 HU; no calcification, mitral-annular continuity, myocardial invasion, or arterial/delayed enhancement; proximal LCx moderate-to-severe stenosis. CE-Echo: no appreciable internal perfusion; later archived images at 1:11 and 2:23 remained nonperfused | — | Edoxaban 60 mg once daily continued | Cancer imaging remained stable | Early-phase CE-Echo cine loops and quantitative peak-intensity ratio unavailable; CMR recommended but declined for financial reasons |
| Nov 26, 2025 | No clinical systemic embolic event reported; serial brain MRI showed no new acute/subacute ischemic lesion suggestive of embolism | TTE: 51.0 × 20.0 × 27.0 mm; increase in maximum linear dimension prompted diagnostic and anticoagulant-exposure reassessment | 41% | Edoxaban 60 mg once daily continued; no major/clinically relevant nonmajor bleeding | Almonertinib continued; cancer imaging stable | Edoxaban-calibrated anti-Xa activity and plasma concentrations not measured; ALT transiently increased to 74 U/L without interruption/reduction |
| Apr 2026 | Alive with NYHA class II symptoms | TTE: 39 × 19 × 29 mm; maximum linear dimension lower than Nov 2025, other two dimensions similar; broad-based/fixed; no internal Doppler flow; LV motion reduced/dyssynchronous | 45% | Edoxaban 60 mg once daily continued | Almonertinib continued; thoracic disease stable | Fractional shortening 22%; no volumetric conclusion drawn from serial linear measurements |

**Abbreviations:** CE-Echo, contrast-enhanced echocardiography; CCTA, coronary computed tomography angiography; CMR, cardiac magnetic resonance; DVH, dose-volume histogram; eGFR, estimated glomerular filtration rate; fx, fractions; INR, international normalized ratio; LAD, left anterior descending coronary artery; LCx, left circumflex coronary artery; LV, left ventricle; LVEF, left ventricular ejection fraction; NYHA, New York Heart Association; PE, pulmonary embolism; RT, radiotherapy; RWMA, regional wall-motion abnormality; TTE, transthoracic echocardiography.

**Supplementary Figure S1.** Serial carcinoembryonic antigen and D-dimer measurements during follow-up. Values measured from February to November 2025 fluctuated without a consistent monotonic trend. CEA, carcinoembryonic antigen.

**Supplementary Figure S2.** Serial brain magnetic resonance imaging findings. (A, D) Axial contrast-enhanced T1-weighted images obtained in February 2023 show enhancing metastases in the left occipital lobe (A, arrow) and brainstem or pontine region (D, arrow). (B, E) Follow-up contrast-enhanced T1-weighted images obtained in November 2025 show marked regression, with no clearly visible residual enhancement at the selected levels. (C, F) Noncontrast axial T1-weighted images obtained in April 2026 show no clear new mass at the corresponding levels. MRI, magnetic resonance imaging.

**Supplementary Figure S3.** Coronary computed tomography angiography showing proximal left circumflex coronary artery stenosis. (A) Three-dimensional volume rendering shows the course of the coronary arteries; the arrow marks the proximal left circumflex coronary artery lesion. (B, C) Multiplanar reconstructions at different angles show focal moderate-to-severe narrowing with a noncalcified plaque. Green markers identify the lesion. CCTA, coronary computed tomography angiography; LCx, left circumflex coronary artery; MPR, multiplanar reconstruction; VR, volume rendering.
